# The Poisonous Properties of Chemically Pure Water

**Published:** 1899-03

**Authors:** 


### *EDITORIAL NOTES AND COMMENTS.*

The Business office of THE JOURNAL is 305, 306 Fitten Building.

The Editorial office is Rooms 401, 402 Grand Opera House.

Address all Business communications to Dr. M. B. Hutchins, Mgr.

Make remittances payable to THE ATLANTA MEDICAL AND SURGICAL JOURNAL.

On matters pertaining to the Editorial and Original communications address Dr. Dunbar Roy, Grand Opera House, Atlanta.

Reprints of original articles will be furnished at cost price. Requests for the same should always be made *on the manuscript*.

We will present, post-paid, on request, to each contributor of an original article, twenty (20) marked copies of THE JOURNAL containing such article.

### THE POISONOUS PROPERTIES OF CHEMICALLY PURE WATER.

THE State has recently been much agitated over the claim of the Milledgeville Water-works that the water would be polluted if the farm bought by the State for the Sanitarium should be occupied and used as intended. Drs. Baird, of Atlanta, and O'Daniel, of Bullards, were appointed by the governor as experts to look into the matter. Their report says that the water would not be polluted. *Apropos* of this controversy, we publish a recent editorial from the *Medical Register*, of Richmond, Va., bearing upon the subject of pure water. It says:

"No more startling proposition could well be conceived than the fact, which has long been known to physiologists, that the drinking of chemically pure water, *i. e.*, water containing no dissolved salts or gases, is actually poisonous to the animal organism. The subject is fully discussed by Hans Koeppe in the *Deutsche Medicinische Wochenschrift* of September 29, 1898. The purity of water is determined by testing its electric conductivity, and it is found that it is almost impossible to secure it absolutely pure. According to this writer, ordinary spring water has a conductivity of 500 to 600 or more on the scale employed, while commercial distilled water has a conductivity of over 49. It is exceedingly difficult to prepare water of less than 2.13. Distilled water is an active protoplasmic poison, due to its property of extracting salts from animal tissues and causing them to swell up by imbibition. When taken into the stomach it causes a swelling of the gastric epithelium, which is followed by desquamation and the production of a catarrhal inflammation. The practice of washing out the stomach with distilled water is condemned, but were it possible to obtain a really pure water the procedure would be even more injurious. The remarkable fact is brought out that there occurs in nature waters purer than ordinary distilled water. Such, it is claimed, is the water from clear natural ice, and it is to this fact that the gastritis produced by giving patients 'ice pills' to allay nausea is said to be attributable. In the guide-books it is customary to warn tourists against quenching their thirst with melted snow and the ice of glaziers. It has been supposed that the danger lay in the temperature of the water from these sources, but, according to Dr. Koeppe, its great purity is the cause of its injurious effects. Melted artificial ice is said to be less harmful. In practical proof of the poisonous qualities of pure water, the writer cites the instance of a spring at Gastein which has for centuries been known as the *Giftbrunnen*, or 'poison spring.' All chemical analyses of this water—and hundreds have been made—have failed to show the slightest trace of any injurious substance, yet it has well merited the name it so long has borne. It now appears that its poisonous qualities are due to its extreme purity! Its electric conductivity is only 31.9, far less than that of ordinary distilled water.

"In view of the facts brought out in the article referred to, a most pertinent question suggests itself: Within recent years the recognition of the dangers lurking in water obtained from natural sources has led to the rather extensive introduction of distilled water for table use, and domestic stills are widely advertised in both medical and lay journals. Now it seems as if the use of such water, while avoiding the danger of imbibing disease germs, exposes the drinker to the equally undesirable alternative of consuming an actual poison. Practically, however, where such water is taken at meal-time it would, we imagine, be free from danger, as it would at once be mixed in the stomach with food rich in salts. For use at other times it would be a simple matter to add to it a sufficient amount of saline material, and, judging from the published analyses of some celebrated 'mineral waters,' the drinking of which is at least not injurious, this addition need be very insignificant in amount."

